# Bacterial Systematic Genetics and Integrated Multi-Omics: Beyond Static Genomics Toward Predictive Models

**DOI:** 10.3390/ijms26199326

**Published:** 2025-09-24

**Authors:** Tatsuya Sakaguchi, Yuta Irifune, Rui Kamada, Kazuyasu Sakaguchi

**Affiliations:** 1 Department of Chemistry, School of Medicine, Kurume University, Kurume 830-0011, Japan; 2 Laboratory of Biological Chemistry, Department of Chemistry, Faculty of Science, Hokkaido University, Sapporo 060-0810, Japan; yuta-irifune@sci.hokudai.ac.jp; 3 Chemistry of Functional Molecules, Graduate School of Biomedical Sciences, Nagasaki University, Nagasaki 852-8521, Japan; rkamada@nagasaki-u.ac.jp

**Keywords:** antibiotic resistance, genome, interactome, machine learning, multi-omics, protein-protein interaction, proteome, transcriptome, quantitative trait loci (QTL)

## Abstract

The field of bacterial systems biology is rapidly advancing beyond static genomic analyses, and moving toward dynamic, integrative approaches that connect genetic variation with cellular function. This review traces the progression from genome-wide association studies (GWAS) to multi-omics frameworks that incorporate transcriptomics, proteomics, and interactome mapping. We emphasize recent breakthroughs in high-resolution transcriptomics, including single-cell, spatial, and epitranscriptomic technologies, which uncover functional heterogeneity and regulatory complexity in bacterial populations. At the same time, innovations in proteomics, such as data-independent acquisition (DIA) and single-bacterium proteomics, provide quantitative insights into protein-level mechanisms. Experimental and AI-assisted strategies for mapping protein–protein interactions help to clarify the architecture of bacterial molecular networks. The integration of these omics layers through quantitative trait locus (QTL) analysis establishes mechanistic links between single-nucleotide polymorphisms and systems-level phenotypes. Despite persistent challenges such as bacterial clonality and genomic plasticity, emerging tools, including deep mutational scanning, microfluidics, high-throughput genome editing, and machine-learning approaches, are enhancing the resolution and scope of bacterial genetics. By synthesizing these advances, we describe a transformative trajectory toward predictive, systems-level models of bacterial life. This perspective opens new opportunities in antimicrobial discovery, microbial engineering, and ecological research.

## 1. Introduction

Bacterial genomes are small, typically ranging from approximately 0.5 to 10 million bases [1]. Nevertheless, they display remarkable plasticity and encode a diverse array of traits that are crucial for medicine, industry, and environmental processes. These traits include antibiotic resistance, virulence, bioproduction, and ecological adaptation [2,3,4,5]. For decades, systematically dissecting the relationship between bacterial genotypes and phenotypes has been a formidable challenge. The complexity of these traits and the unique characteristics of microbial population genetics, such as clonality and pervasive linkage disequilibrium, have posed significant barriers [6]. Recently, however, the field has been undergoing a notable transition. Methodological advances are driving bacterial genetics beyond simple correlational studies toward mechanistic, systems-level analyses. These approaches hold the promise of uncovering the intricate molecular architecture of bacterial cells [7,8,9].

**Figure 1:**
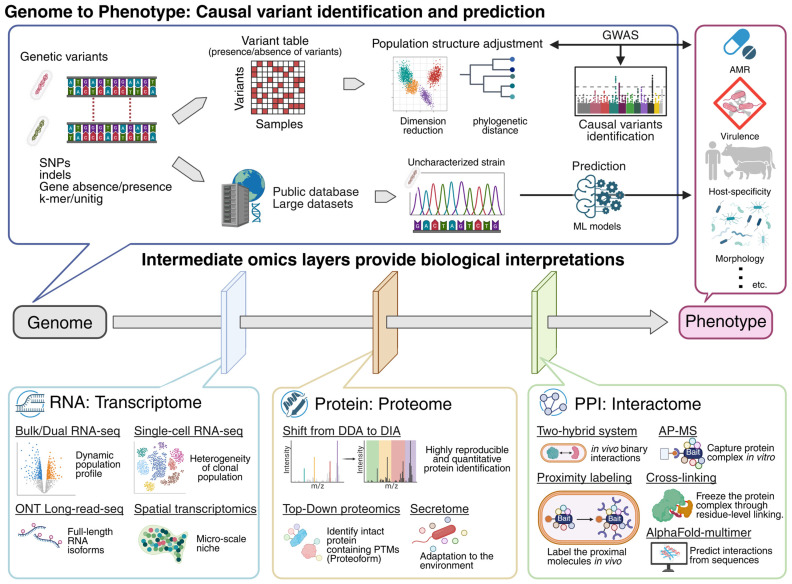
Overview of bacterial omics layers and recently advanced technologies. The schematic diagram illustrates the conceptual workflow connecting bacterial genomic variation to complex phenotypes through multi-omics integration. (**Top**) Starting from the genome, diverse genetic features, including single-nucleotide polymorphisms (SNPs), insertions/deletions (indels), gene presence–absence patterns, and k-mers/unitigs, are extracted to represent the plasticity of bacterial genomes. Statistical frameworks such as genome-wide association studies (GWAS), often incorporating population structure adjustment (e.g., phylogenetic distances, dimensionality reduction), identify causal variants by associating them with diverse bacterial phenotypes. In parallel, extensive genomic and phenotypic datasets are stored in public databases and used to train machine-learning (ML) models for predicting phenotypes directly from genomic sequences. (**Bottom**) Intermediate omics layers provide mechanistic interpretation: transcriptomics (bulk, dual, single-cell, spatial) reveals dynamic and heterogeneous gene expression; proteomics (data-independent acquisition, top-down proteomics, single-bacterium proteomics, secretomics) quantifies protein abundance and post-translational modifications; and interactome mapping (two-hybrid systems, affinity purification, proximity labeling, cross-linking, structural prediction) elucidates molecular interaction networks. Integration of these layers enables causal inference linking genetic variants to system-wide molecular changes and phenotypic traits such as antimicrobial resistance, morphology, host specificity, and virulence.

This review explores the expanding frontier of biological inquiry by charting the path from genetic variation to complex functional cellular networks [3,5,10,11] (Figure 1). We begin by examining genome-wide association studies (GWAS), and machine learning approaches that have substantially enhanced the accuracy of phenotype prediction directly from genomic sequences [6,8,12,13]. While these methods are powerful, they often fall short of fully explaining the underlying biological mechanisms. To bridge this gap, we next explore intermediate omics layers that provide essential functional context [7,14,15,16,17,18].

Transformative advances in transcriptomics, including single-cell, spatial, and dual RNA sequencing, now reveal the dynamic and heterogeneous nature of gene expression [19,20,21,22,23,24,25]. We next examine the proteomic landscape, emphasizing progress in high-throughput quantitative mass spectrometry, which enables precise measurements of the cell’s functional machinery, from individual proteoforms to the complete secretome [17,18,26,27].

Since biological function emerges from molecular interactions, we also discuss the bacterial interactome: the network of protein–protein interactions that orchestrate cellular processes [28,29,30,31]. Sophisticated methodologies, including genetic screens, affinity purification, proximity labeling, and cross-linking mass spectrometry, are now enhanced by the predictive power of artificial intelligence. Finally, we discuss how these diverse data layers are increasingly integrated within the framework of quantitative trait locus (QTL) mapping [10,32,33].

The identification of expression-level (eQTL) and protein-level (pQTL) associations provides direct mechanistic links between genetic variation and molecular function. These molecular QTLs serve as the foundation for reconstructing the regulatory and metabolic networks that govern bacterial life, ultimately realizing the long-envisioned “from SNPs to networks” framework. By reviewing these innovations and their diverse applications, we aim to provide a comprehensive overview of the current state of bacterial systems genetics and to illuminate its transformative trajectory.

## 2. Recent Studies of Bacterial GWAS, Intermediate Molecular Omics, and Multi-Omics Integration

The transition from bacterial genome sequencing to a comprehensive understanding of phenotypic traits has been driven by continuous advancements in analytical methodologies. Genome-wide association studies (GWAS) have been pivotal in revealing statistical relationships between genetic variants and observable phenotypes. In recent years, the integration of large-scale datasets with advanced computational approaches, including machine learning, has improved the identification of causal genes and enabled increasingly accurate phenotype predictions directly from genomic sequences.

Despite these advances, predictive approaches often fail to distinguish correlation from causation [10]. To address this limitation, recent studies have focused on intermediate molecular layers. Systematic profiling of the transcriptome and proteome captures the dynamic functional states of cells, bridging the gap between the static genome and the emergent complexity of phenotypes. In this review, we first summarize recent progress in bacterial GWAS and then highlight advances in intermediate-layer omics, which complement and refine GWAS-based strategies.

### 2.1. Genome-Wide Association Studies

The growing availability of bacterial whole-genome sequences has substantially advanced our understanding of genotype–phenotype relationships. Comprehensive databases now integrate genomic information with detailed phenotypic annotations, especially for clinically relevant traits such as antibiotic resistance. This rapidly expanding resource has driven the application of bacterial GWAS, enabling the identification of genetic determinants linked to diverse phenotypic traits [3,5]. GWAS has become a key approach for uncovering genetic variants underlying specific phenotypes, providing a hypothesis-free framework for genome-wide scanning [34]. However, implementing GWAS in bacterial systems presents unique challenges and necessitates tailored analytical approaches to address bacteria-specific complexities.

A major challenge in bacterial GWAS is the pronounced population structure of bacterial species [4,5]. Since bacteria primarily reproduce clonally, their genomes exhibit strong population structure and tend to be in genome-wide linkage disequilibrium (LD). This means that genetic variants, including mutations, are frequently inherited together as large blocks or across the entire chromosome. Consequently, variants that are neutral or not directly causal to the phenotype of interest may be falsely identified as causal. To address this issue, linear mixed models (LMMs) are commonly used. LMMs incorporate a genetic similarity matrix, which quantifies genetic relationships or similarities among samples, as a random effect. This matrix is derived from various genetic variants, or phylogenetic distances. This methodological framework enables LMMs to assess the phenotypic influence of individual loci within the broader genomic context, thereby reducing the confounding effect of loci strongly associated with the underlying population structure and allowing more accurate identification of phenotype-associated loci. Several tools implement LMMs for bacterial GWAS, including GEMMA [35], Pyseer [36], and Bugwas [37]. Dimensionality reduction techniques, such as principal component analysis (PCA) and multidimensional scaling (MDS), are also used to capture genetic variation associated with population structure [6]. HAWK, for instance, performs PCA on the presence–absence matrix of k-mer [38,39], whereas Pyseer corrects for clonal population structure using MDS on a distance matrix [36].

Another major challenge in bacterial GWAS is the pronounced plasticity of the bacterial genome [40]. Bacterial genomes are organized in a pan-genomic manner, comprising core genes shared by all strains and accessory genes that vary among strains [41]. Even strains with identical gene content can differ substantially in gene order and overall genome architecture, which limits the effectiveness of reference-based alignment. To capture this genomic diversity, bacterial GWAS incorporates multiple types of genetic features, including single-nucleotide polymorphisms (SNPs), insertions/deletions (indels), gene presence–absence patterns, k-mers, and aggregated forms known as unitigs [3,4,5]. Among these, k-mers and unitigs are particularly well suited for bacterial GWAS because they comprehensively represent pan-genomic variation [8,9,12,42]. A k-mer is a contiguous nucleotide sequence of fixed length *k* that represents all overlapping subsequences within a DNA sequence. A unitig is a contiguous sequence constructed by aggregating overlapping k-mers across samples within a de Bruijn graph (DBG). Unitigs preserve the genomic signal of their constituent k-mers while providing a compact and interpretable representation across samples. An important advantage of both k-mers and unitigs is that they do not require a reference genome and can be applied directly to raw sequencing reads [43]. Because of these advantages, k-mer- and unitig-based approaches have been implemented in numerous bacterial GWAS tools, such as HAWK [38,39], GEMMA [35], Bugwas [37], Pyseer [36], DBGWAS [44], and Scoary [45,46].

Recent innovations have extended bacterial GWAS beyond antimicrobial resistance to include traits such as pathogenicity [47,48], metabolic capabilities [49], biofilm formation [50,51], and host specificity [52,53,54]. For example, Boeck et al. analyzed 331 clinical *Mycobacterium abscessus* isolates, identifying both well-known resistance-associated mutations—such as those in 16S and 23S rRNA genes linked to aminoglycoside and macrolide resistance—and novel missense mutations associated with intracellular replication, a complex and multifactorial trait [55]. Yang et al. reviewed bacterial GWAS studies across more than 20 species, spanning Gram-positive and Gram-negative bacteria, pathogenic and commensal organisms, and diverse ecological niches. Their review drew on insights from over 200 original studies [5].

Most bacterial GWAS studies involve sample sizes of several hundred to a few thousand isolates and show robust performance in identifying causal variants [3,4,5]. The advent of large-scale genomic datasets has also enabled the development of highly accurate and generalizable predictive models [56,57,58,59,60]. A notable example is the Comprehensive Resistance Prediction for Tuberculosis: an International Consortium (CRyPTIC) study of Mycobacterium tuberculosis, which analyzed whole-genome sequences from more than 10,000 clinical isolates with binary susceptibility data for four first-line tuberculosis antibiotics [56]. This project produced a comprehensive catalog of resistance-associated genes and developed a robust, interpretable model capable of predicting resistance to four first-line tuberculosis antibiotics with 90–95% accuracy. Its high interpretability and clinical applicability highlight the potential of genome-informed phenotype prediction to advance microbiology toward actionable, model-driven understanding.

Integration of large-scale datasets into unified databases has become essential for phenotype prediction [43,61,62,63,64]. BacDive is among the largest curated prokaryotic resources, containing genomic and phenotypic data for over 97,000 strains and more than 2.6 million individual data points spanning a wide range of physiological traits [61]. To leverage this diversity, Koblitz et al. developed random forest-based prediction models accessible via BacDive, achieving over 96% accuracy for key bacterial traits such as Gram stain, spore formation, flagellation, aerobic or anaerobic metabolism, and thermophily [13]. Similarly, Hyun et al. used support vector machine (SVM) models to predict antimicrobial resistance (AMR) across 12 bacterial species. Their dataset included 27,155 genomes with AMR metadata for 69 antibiotics from the PATRIC database [59,62]. This approach identified 263 known AMR genes, nearly twice the number recovered using Pyseer (145 genes), and proposed 142 novel AMR gene candidates. However, the complexity of machine learning approaches complicates biological interpretation [12]. For instance, a deep learning model for AMR prediction achieved high accuracy even without known resistance genes, suggesting minimal reliance on previously characterized markers [65]. The limited interpretability of such models remains a key barrier to elucidating the molecular basis of phenotypic traits. Thus, integrating tools that seek to explain the decision-making process of the AI models, such as SHAP (Shapley Additive Explanations) [66], LIME (local interpretable model-agnostic explanations) [67], and a preference for transparent algorithms, should be pursued. This will not only foster a deeper understanding of the underlying mechanisms but also build trust and confidence in the predictive models [68].

### 2.2. Transcriptome

Genomic information provides insight into the potential capabilities of a bacterium, but it represents only a static blueprint. Transcriptomics serves as a critical bridge, linking these static genetic instructions to the dynamic physiological states of bacterial cells [7,69,70]. Foundational work in this field relied on bulk RNA sequencing (RNA-seq), which provides an averaged snapshot of gene expression across a cell population. Recent technological advances have greatly expanded the resolution and interpretive power of bacterial transcriptomics. Long-read sequencing now resolves complete operons, clarifying transcriptional units and their regulatory architecture [71,72]. Single-cell transcriptomics enables profiling at the level of individual cells, revealing previously hidden heterogeneity [21,22,23,24]. Spatial and probe-based approaches further enrich this view by visualizing gene expression in native microenvironments, providing insights into bacterial interactions, niche adaptation, and disease processes [25,73,74]. These innovations have substantially expanded analytical capabilities, allowing for deeper biological insights into regulatory network organization, transcriptional dynamics, and host–pathogen interactions [20,75,76].

Bulk RNA-seq remains an indispensable tool for global transcriptome profiling. Its high throughput allows researchers to survey hundreds of conditions or isolates, generating broad overviews of gene activity and identifying transcriptomic signatures of biological processes and disease mechanisms [7,14,69]. Dual RNA-seq has further transformed the study of host–microbe interactions [75,76]. By sequencing both host and microbial transcripts simultaneously, this approach dissects infection dialogues and cross-kingdom communication. For example, in a mouse model of brain abscess, dual RNA-seq revealed that C3 signaling reshaped the *Staphylococcus aureus* stress regulon and triggered a microglial inflammatory response, providing mechanistic insights with therapeutic implications [20]. Comparative transcriptomics also illuminates adaptive trade-offs [7,69]. Ryan et al., for instance, generated a high-resolution transcriptome atlas for *Bacteroides thetaiotaomicron* across 15 environmental conditions, including variations in carbon source, pH, oxygen, heat stress, and tetracycline exposure. This analysis identified approximately 300 regulons and more than 140 small RNAs, highlighting the flexibility of transcriptional networks [77].

Oxford Nanopore Technology (ONT) has driven major advances in bacterial transcriptomics. Long-read sequencing enables precise mapping of transcript boundaries [71]. For example, Tan et al. extended 225 previously annotated operons in *Escherichia coli* and 89 in *Staphylococcus aureus* [72]. ONT’s direct RNA sequencing (DRS) additionally detects RNA modifications, such as N6-methyladenosine (m6A) [71,72,78]. This approach has elucidated ribosomal RNA maturation by capturing intermediate forms [79] and revealed RNA modification-mediated regulatory mechanisms under heat stress [80]. Despite these advantages, long-read sequencing and DRS face limitations in bacterial systems, particularly in throughput and base-calling accuracy [81,82]. Nevertheless, their ability to sequence full-length native RNA molecules while preserving secondary structures and chemical modifications remains a unique strength. As these technologies mature, they are expected to play an increasingly central role in mapping the bacterial transcriptome and epitranscriptome.

A major limitation of bulk RNA-seq is its population averaging, which masks cell-to-cell heterogeneity [83,84]. Early bulk RNA-seq studies premised that genetically identical bacteria respond uniformly to environmental cues, but single-cell approaches have revealed profound variability. MATQ-seq pioneered bacterial single-cell RNA-seq (scRNA-seq) by combining fluorescence-activated cell sorting (FACS) with random-hexamer priming. It captured, on average, 170 genes per *Salmonella typhimurium* cell and 102 per *Pseudomonas aeruginosa* cell, revealing growth-condition-specific subpopulations and pronounced transcriptional heterogeneity [85]. It unveiled growth-condition-specific subpopulations and transcriptional heterogeneity. In addition, optimized MATQ-seq boosted capture efficiency, making MATQ-seq suitable for hundreds of samples, a distinct advantage over droplet methods [23]. PETRI-seq, using a three-round split-pool ligation strategy, profiled tens of thousands of cells and detected a 0.04% prophage-induced minority in wild-type *S. aureus* cultures [21]. Using this method, Poutain et al. demonstrated the effect of chromosomal replication and classified genes according to their transcription–replication interaction profiles (TRIPs) [70]. MicroSPLiT, another split-pool method, extended profiling to more than 25,000 Bacillus subtilis cells across growth phases, identifying 14 clusters, including a rare subpopulation representing only 0.142% of cells (36 cells) [22]. Such rare subpopulations, potentially involved in bet-hedging strategies [86,87], cannot be captured reliably by bulk or low-throughput approaches. Microfluidic droplet-based platforms have further increased throughput. M3-seq, which combines combinatorial indexing with post hoc RNase H–mediated rRNA depletion, profiled tens of thousands of cells per run [88]. This revealed a pre-existing *E. coli* subpopulation that constitutively expresses the acid-resistance regulon and rapidly dominates after pH shock, providing direct evidence of stochastic pre-adaptation. By applying universal rRNA depletion, BacDrop uncovered coexisting transcriptional programs associated with persister formation, mobile element activation, and SOS repair, thereby explaining heterogeneous drug susceptibility in *Klebsiella pneumoniae* treated with ciprofloxacin [24]. smRandom-seq, which uses CRISPR–Cas9–mediated rRNA cleavage instead of RNase H, routinely captures ~1000 genes per cell and has identified rare *E. coli* cells that hyperactivate the SOS network to initiate plasmid-borne resistance before the bulk population detects DNA damage [89]. Probe-based methods add another layer of resolution. ProBac-seq uses DNA probe libraries to hybridize with specific mRNAs in fixed cells for targeted and high-sensitivity profiling. Using commercial droplet platforms, ProBac-seq resolved *B. subtilis* subpopulations and detected acetate-responsive toxin expression in *Clostridium perfringens*, demonstrating its utility for precise pathogenic gene expression studies [73].

Transcript counts alone cannot explain community behavior without spatial context. par-seqFISH imaging of 105 marker genes in *Pseudomonas aeruginosa* microcolonies revealed micron-scale metabolic zoning [25]. For example, this approach uncovered functional compartmentalization within single microaggregates, where distinct regions exhibited activities such as survival metabolism, virulence factor biosynthesis, and energy state regulation. While spatial information at the community level is essential, Sarfatis et al. extended this concept to the intracellular scale. Their Bacterial-MERFIS technique achieves approximately 1000-fold volumetric expansion of individual cells by embedding them in a robust expansion gel [90]. By analyzing 296,666 *E. coli* cells across 1057 operons, they uncovered heterogeneous responses to carbon source availability. Their findings suggest a stochastic, hierarchical progression along carbon utilization operons, triggered by glucose deprivation, indicating a diversification strategy in response to carbon starvation [90].

### 2.3. Proteome

Transcriptomics illuminates the dynamic regulatory landscape linking the genome to cellular activity. However, because proteins are the principal effectors of biological processes, direct proteome analysis is essential for a mechanistic understanding of cell function. Proteomics provides quantitative insights into protein abundance, post-translational modifications (PTMs), and the organization of proteins within macromolecular complexes [16,91,92]. As catalysts, transporters, signaling mediators, and structural scaffolds, proteins execute nearly all cellular processes. Consequently, proteome-level data provide a more immediate link between genotype and phenotype than either the genome or transcriptome alone [93]. In microbiology, proteomics has advanced well beyond simple protein cataloging. Comparative and metaproteomic studies have revealed protein-level strategies underlying antibiotic resistance—such as target protection, drug inactivation, efflux, and stress acclimation—across diverse bacterial pathogens [18,94]. Community-scale surveys have further shown how bacterial proteomes reorganize in response to cultivation conditions, nutrient regimes, and environmental gradients, yielding functional maps of microbial ecosystem services [95]. This section highlights key advances and emerging technologies in bacterial proteomics, including data-independent acquisition (DIA), top-down proteomics (TDP), and single-cell or spatially resolved approaches. Together, these innovations are transforming our ability to interrogate bacterial proteomes in situ and to translate proteomic insights into clinical and environmental applications.

Bottom-up proteomics, in which proteins are enzymatically digested into peptides prior to mass spectrometry, remains the standard for large-scale protein identification and quantification [27]. Traditional workflows rely on data-dependent acquisition (DDA), where the instrument first performs a survey scan to detect peptide precursor ions and then fragments only the most intense ions. This stochastic selection leads to missing values because low-abundance peptides are often skipped when higher-intensity signals dominate. To overcome this limitation, the field is increasingly adopting data-independent acquisition (DIA), which systematically fragments all detectable precursor ions, yielding more comprehensive and reproducible peptide profiles [27,96]. In DIA workflows, the instrument cycles through predefined mass-to-charge (*m*/*z*) windows and fragments all ions within each window regardless of intensity. Benchmarking studies using a defined 12-member microbial mock community demonstrated that DIA matched or exceeded DDA in proteome coverage, quantitative accuracy, and cross-laboratory reproducibility [96]. The reliability of DIA-MS has enabled absolute protein quantification, including determination of copy numbers for thousands of proteins per cell, which is critical for building system-level models of bacterial physiology [97].

Top-down proteomics (TDP) analyzes intact proteins, enabling direct observation of “proteoforms”, defined by specific genetic variants and post-translational modifications [98,99,100]. This approach moves beyond primary sequences to characterize functionally distinct molecular species.

Historically, TDP was limited to purified proteins or simple mixtures due to challenges in intact protein separation, ionization, and fragmentation. Recent advances have overcome some of these barriers. For example, Dupré et al. identified approximately 220 proteins and more than 500 proteoforms from *Escherichia coli*, revealing strain-specific proteoforms across diverse bacterial genera. Their work enabled proteoform-based phylogenetic analyses and enhanced genome annotations [101]. Additionally, a capillary-based method combined with TDP allowed for the detection of over 200 proteoforms from just 50 pg of intact *E. coli* lysate [102]. Unlike bottom-up proteomics, TDP distinguishes unmodified and modified protein species, providing unique insight into active molecular forms. This proteoform-level resolution is valuable for diagnostic development and for identifying new therapeutic targets.

Proteomics is now extending toward single-cell resolution, paralleling developments in transcriptomics. This approach allows the detection of rare, functionally important subpopulations that are invisible in bulk analyses [103]. Extending single-cell proteomics to bacteria, however, is particularly challenging because a single *E. coli* cell is roughly 1000-fold smaller than a mammalian cell and contains only femtograms of protein. A breakthrough in single-bacterium proteomics (SBP) came from Végvári et al., who achieved protein detection in individual *E. coli* cells [104]. Their approach adapted SCOPE-MS (Single Cell ProtEomics by Mass Spectrometry) [26] for bacteria. SCOPE-MS uses a “carrier proteome”, comprising lysate from 100 to 200 cells that is isobarically labeled and multiplexed with single-cell samples. The carrier boosts peptide abundance to trigger MS/MS fragmentation while preserving quantitative detection of reporter ions from the single-cell peptides. Using this strategy with advanced mass spectrometry, Végvári et al. identified over a dozen proteins from individual bacterial cells and distinguished between samples containing one versus two cells. This proof-of-concept demonstrates that SBP can uncover heterogeneity at the single-bacterium level.

Bacterial proteomics encompasses both intracellular and extracellular proteins. Bacteria secrete enzymes that harvest nutrients, adhesins that promote biofilm formation, signaling molecules for quorum sensing, and effectors that remodel host physiology [105]. Secretome analyses have been performed in species such as *P. aeruginosa* [106] and *Mycoplasma* [107], but secretory proteins remain technically challenging to study because they are often present at extremely low abundance. Secreted proteins are typically several orders of magnitude less concentrated than their intracellular counterparts. Moreover, extracellular media often contain salts, polysaccharides, and host-derived proteins, which can obscure low-abundance bacterial peptides. Traditional concentration methods, such as ultrafiltration, precipitation, and dialysis, require large volumes and often result in substantial sample loss. As a result, these methods are unsuitable for high-throughput applications. To address this, Russo et al. developed EXCRETE (Enhanced eXoproteome ChARacTERization), a 96-well-compatible protocol that integrates one-pot reduction and alkylation, bead-based aggregation, and on-bead digestion for same-day LC-MS analysis [108]. Applied to three cyanobacterial species, EXCRETE identified an average of 3974 peptides and 639 proteins per species, approximately double the yield of ultrafiltration. The study revealed that cyanobacterial secretomes are enriched in cell-envelope and nutrient-uptake proteins, challenging the long-standing assumption that cyanobacteria are largely non-secretory.

A comprehensive bacterial proteomic resource now includes 303 species, 119 genera, and five phyla, comprising more than 636,000 unique expressed proteins. This dataset is publicly available through ProteomicsDB [17]. Using this resource, the MS2Bac algorithm identifies bacterial strains by iteratively querying the NCBI bacterial proteome database, achieving over 99% accuracy at the species level and 89% at the strain level.

The bacterial transcriptomics and proteomics technologies discussed in this review are summarized in Table 1.

### 2.4. Interactome

Proteomics provides a quantitative catalog of the cell’s functional machinery, but cellular function is rarely explained by single proteins in isolation. Instead, biological processes emerge from a complex, dynamic, and context-dependent network of molecular interactions [109]. This comprehensive map of molecular connections, known as the interactome, underpins the regulation and execution of nearly all cellular activities [110]. Systematic characterization of the interactome is therefore essential for transforming an inventory of cellular components into a mechanistic model of the cell as an integrated system. Protein–protein interactions (PPIs) are central to bacterial physiology and represent an emerging class of antibiotic targets [30]. Furthermore, bacterial interactomes are highly dynamic, responding rapidly to environmental fluctuations [111]. For example, in the bacterial RNA-binding proteome, nearly 70% of RNA-binding proteins showed significant changes in RNA-binding activity across growth phases, revealing a critical layer of post-transcriptional regulation [112]. This dynamic and modular nature facilitates rapid adaptation to environmental changes. Bacterial protein–protein interactions (PPIs) are not only fundamental to cellular physiology but also represent an emerging class of antibiotic targets [113]. Many essential processes rely on multiprotein complexes, and the disruption of a single component can be lethal [114,115]. Interaction interfaces within these complexes, therefore, constitute a largely untapped reservoir of pharmacologically tractable sites. In addition, PPIs mediate critical bacteria–host interactions, offering opportunities for next-generation antibacterial strategies [116,117,118].

Binary PPI mapping often relies on two-hybrid systems, in which a reporter protein is split into two fragments fused to “bait” and “prey” proteins [119]. Interaction of the bait and prey reconstitutes the reporter and generates a detectable signal. While the yeast two-hybrid (Y2H) system [120] is well used, the bacterial two-hybrid (B2H) system [121] provides a more native environment for bacterial proteins. B2H is particularly effective for membrane-associated proteins and shows a lower rate of autoactivation-driven false positives [122]. A comprehensive B2H screen of the *Legionella pneumophila* effector proteome challenged the view that these virulence factors act independently. The study revealed numerous effector–effector interactions, uncovering a previously hidden regulatory network and expanding the concept of metaeffectors, defined as effectors that modulate the function of other effectors in host cells [28].

Affinity purification–mass spectrometry (AP-MS) complements genetic approaches by isolating multiprotein complexes from cell lysates. Tagged “bait” proteins are used to capture their interacting partners, which are then identified by mass spectrometry [29,123]. Although early AP-MS studies suffered from high background contamination [30], advances such as quantitative AP-MS (q-AP-MS) and robust scoring algorithms have greatly improved specificity [29,124,125]. The potential of q-AP-MS is exemplified by the mapping of the *Mycobacterium tuberculosis* (Mtb)–human interactome. This study revealed that the Mtb virulence factor LpqN binds the human E3 ubiquitin ligase CBL, and functional assays demonstrated that this interaction suppresses host antibacterial activity [126]. Proximity labeling (PL) further extends interactome mapping by capturing weak or transient interactions in live cells [127,128,129]. A promiscuous labeling enzyme fused to the bait generates short-lived reactive species that covalently tag nearby proteins (typically within 10–20 nm) [130]. Labeling occurs before cell lysis, preserving interactions that would otherwise be lost and minimizing post-lysis artifacts [31]. PL has been successfully adapted to bacteria; for example, optimized protocols now allow the detection of stable and transient interactomes in *Myxococcus xanthus* [131].

Cross-linking mass spectrometry (XL-MS) adds a structural dimension to interactome studies by providing distance constraints between amino acid residues [132]. Chemical cross-linkers covalently link spatially proximal residues, and mass spectrometry identifies cross-linked peptides to reveal residue-level proximity. Conducting cross-linking in vivo can effectively “freeze” transient interactions [133]. The distance constraints from XL-MS are invaluable for modeling the topology of large assemblies and refining computational structural predictions. This approach has provided insights into bacterial molecular machines that are challenging for conventional structural biology, including the ribosome and the flagellar motor [134]. A pioneering in vivo XL-MS study of human cells infected with *Acinetobacter baumannii* directly identified cross-links between bacterial virulence factors and human targets, yielding the first structural information on these host–pathogen interfaces [135].

Computational methods have revolutionized and accelerated the development of interactomics. AlphaFold-Multimer can now predict the structures of protein complexes from sequence with high speed and accuracy [113]. This capability enables proteome-scale computational screening for PPIs [136,137], and its strength lies in synergy with experimental validation. For example, a novel interaction between two hypothetical proteins in *Bdellovibrio bacteriovorus* was predicted with high confidence by AlphaFold and subsequently confirmed by B2H [138]. Such combined approaches are accelerating the definition of “essential interactomes” in bacteria, providing structural blueprints for the development of novel antibiotics [113]. The rapidly expanding volume of interaction data is increasingly supported by public repositories. The STRING database integrates experimental evidence, genomic context, and text-mined literature to generate confidence-scored networks of physical and functional associations [139]. BioGRID offers a curated collection of physical and genetic interactions, all supported by direct experimental evidence [140]. Additional specialized resources, including PrePPI [141], HVIDB [142], and iPPI-DB [143], further extend coverage of bacterial interactions. Effective use of these databases requires cross-referencing multiple sources and, when possible, consulting the original literature.

The methods used to identify bacterial interactomes discussed in this review are summarized in Table 2.

### 2.5. Bacterial Multi-Omics and QTL Analysis

While interactome mapping yields critical insights into bacterial physiology, a comprehensive systems-level understanding requires the integration of these interaction networks with additional layers of biological information. The development of multi-omics approaches, including genomics, transcriptomics, and proteomics, is beginning to bridge the gap between genetic potential and functional outcomes in bacteria. However, multi-omics integration requires large, high-quality omics datasets under consistent conditions, bacterial integrative field remains in its early stages [9,145,146].

Recent studies highlight the power of integrating multiple omics datasets to uncover fundamental biological principles. A landmark study by Balakrishnan et al. demonstrated that, in *Escherichia coli*, protein abundance is primarily determined by promoter activity [97]. By combining transcriptomic and proteomic data, the study revealed that gene expression regulation is largely independent of fluctuations in shared cellular machinery. They observed that RNA polymerase activity closely matches the cell’s translational output and that most mRNAs display similar translational characteristics. This work provides a quantitative framework to predict protein levels from promoter activity, enabling the inference of gene regulation from multi-omics data.

The opportunistic pathogen *S. aureus* has served as a model for multi-layered network analysis. In one study, researchers developed a novel proximity-labeling approach to map the host–pathogen interactome by decorating the bacterial surface with an enzyme that biotinylates host proteins upon contact. This proteomic strategy identified an endothelial cell surface interactome comprising 305 proteins, including several previously unknown co-receptors involved in *S. aureus* internalization [147]. In a separate study integrating genomics and phenomics, Yang et al. performed a genome-wide association study (GWAS) on 99 *S. aureus* strains to dissect the genetic basis of phenotypic plasticity in response to vancomycin stress and coexistence with *E. coli* [145]. This analysis generated multilayered genetic networks linking specific genetic variants to changes in growth dynamics, providing a systems-level framework for understanding environment-induced evolution.

Extending from single species to complex microbial communities, multi-omics is essential for understanding ecosystems such as the gut microbiome. As highlighted in a recent review by Pinto and Bhatt, advances in high-throughput sequencing now enable integrated analyses at the genomic (metagenomics), transcriptional (metatranscriptomics), and translational (metaproteomics) levels [9]. This approach moves beyond simple compositional surveys toward elucidating the functional roles of microbial communities in health and disease [148].

Integrated multi-omics datasets are large, heterogeneous, and multidimensional, making them well-suited to machine learning. In *E. coli*, Kim et al. developed “Ecomics”, a normalized, quality-controlled compendium from literature and public databases, and built models that predicted genome-wide metabolite concentrations and growth dynamics using four omics layers [149]. Notably, they found that integrating multiple layers consistently outperformed single-layer predictors. Extending this concept, Bi et al. curated a multi-omics knowledgebase for *Bacillus subtilis* and integrated 34 machine learning models with a metabolic network model, enabling accurate prediction of 605 gene expression profiles and synthesis trends for 23 metabolites under diverse growth conditions [150]. Together, these studies illustrate how machine learning can transform complex multi-omics resources into predictive frameworks that can capture non-obvious details and patterns, and use this information to forecast cellular behavior.

Whole-cell models represent the most comprehensive form of multi-omics integration, capturing the activity of nearly every molecule in a bacterial cell [151]. Parameterized with diverse datasets, spanning omics layers, enzyme kinetics, biomolecular half-lives, and intracellular concentrations, these models enable system-level predictions. A landmark example, the *Mycoplasma genitalium* model by Karr et al., revealed new kinetic relationships and guided experimental discoveries [152]. This approach has since been scaled to *E. coli*, predicting complex phenotypes such as protein half-lives and tRNA aminoacylation mechanisms [153,154], and extended to colony-level simulations of antibiotic resistance emergence [155]. These advances underscore that integrating multi-layer biological information is essential for decoding and predicting complex bacterial behavior.

Quantitative trait locus (QTL) mapping is a powerful statistical framework for linking genetic variants to quantitative phenotypic traits. While it is a cornerstone of eukaryotic genetics, its direct application to bacteria has proven challenging. The primary reason is that bacteria reproduce clonally, generating strong population structures and extensive linkage disequilibrium (LD), in which genetic variants are inherited in large blocks [6]. This makes it statistically difficult to pinpoint which specific variant in a block is responsible for a given trait. A particularly tractable application has emerged in the study of host–microbe interactions. Microbiome QTL analyses aim to identify host genetic variants that influence the abundance of specific microbes in the host microbiome [32]. These studies, a form of microbiome genome-wide association study, have successfully linked host genetic variation to the composition of resident microbial communities, offering insights into the co-evolutionary history of host–microbiome relationships. Bacterial GWAS has also been applied to identify bacterial genetic variants associated with key phenotypes such as virulence and host specificity [5]. For example, a pioneering bacterial GWAS in *Campylobacter* revealed that the vitamin B5 biosynthesis pathway is a major genetic determinant of host specificity—a discovery that would have been difficult to achieve using traditional methods [156]. These studies collectively underscore the potential of linking bacterial genotypes to complex phenotypes, provided that the substantial statistical challenges can be addressed.

## 3. Current Challenges and Promising Technologies

Significant progress has been made in bacterial systems biology, yet several fundamental challenges continue to impede the comprehensive mapping and mechanistic understanding of bacterial multi-layer omics and their genetic foundations. A new generation of technologies is emerging to overcome these barriers, setting the stage for unprecedented discovery in bacterial systems biology.

The primary obstacle for true bacterial QTL mapping is the difficulty in generating suitable mapping populations [6]. Unlike yeast, which can be crossed and inbred to break down linkage disequilibrium, bacteria reproduce clonally, resulting in strong population structures that confound genetic association studies [33]. Innovative strategies are beginning to address this challenge. In a landmark study, Vasileva et al. performed iterative rounds of genome shuffling between two *Bacillus subtilis* parental strains, followed by isolation and resequencing hundreds of progenies. This approach generated the first bacterial population suitable for high-resolution QTL mapping [157]. Using this population, they mapped loci controlling complex quantitative traits such as spore germination efficiency and swarming motility, achieving approximately 10 kb resolution. Although still large linkage blocks remained, the study provided a proof of concept for bacterial systems genetics. Complementary approaches involve deep-mutational scanning, including Tn-seq [158] and genome-wide CRISPR/Cas9 libraries [159], which introduce quasi-random mutations across the bacterial chromosome. These mutant populations have minimal population structure, providing an ideal resource for high-resolution QTL mapping. The main remaining hurdle is linking each mutant to reliable genotype–phenotype readouts. Microfluidic technologies are expected to resolve this limitation by enabling automated, high-throughput isolation and single-cell analysis [160]. Microfluidics allows precise control over the cellular microenvironment, including chemical gradients and physical constraints, and supports large-scale phenotyping of genetically diverse populations [161]. Integration with automated imaging generates quantitative datasets on growth, motility, and stress resistance for thousands of lineages in parallel, creating the foundation for accurate QTL mapping.

Conventional interactome methods such as bacterial two-hybrid (B2H) and affinity purification–mass spectrometry (AP-MS) have been invaluable, yet they often require heterologous expression or fail to capture transient interactions. Protein-fragment complementation assays (PCAs) offer a powerful alternative, enabling the detection of protein–protein interactions (PPIs) in vivo under native regulatory control [144,162]. In PCA, a reporter enzyme, such as dihydrofolate reductase or luciferase, is split into two nonfunctional fragments, which are fused to the proteins of interest. When the proteins interact with each other, the fragments refold into an active enzyme that produces a measurable signal. This strategy has generated genome-scale PPI maps in yeast by inserting PCA fragments into endogenous loci [163]. The major barrier to extending PCA to bacteria has been the lack of efficient, high-throughput genome editing. Systematically tagging every bacterial gene with PCA fragments is a formidable task. Recent advances in CRISPR–Cas9 genome editing are beginning to overcome this challenge [164]. Cas9-mediated double-strand breaks can be repaired using donor DNA carrying PCA tags, and unedited sequences can be selectively removed through negative selection, enriching for correctly modified cells. As high-throughput genome editing platforms continue to mature, the construction of genome-scale PCA libraries in bacteria is becoming feasible. This advancement will allow dynamic, in vivo mapping of entire bacterial interactomes under diverse environmental conditions, offering unprecedented systems-level insights into bacterial physiology, adaptive strategies, and novel targets for antimicrobial intervention.

**Figure 2:**
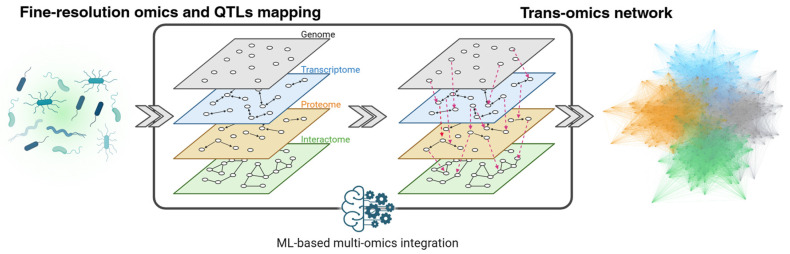
From fine-resolution omics and QTLs to a trans-omics network. Genetically diverse bacterial libraries enable high-resolution omics profiling and QTL mapping. Each omics layer is used to define within-layer networks (**left**). Integrating these layers reveals cross-omics associations ((**right**); dashed magenta arrows), including eQTLs, pQTLs, and interactome QTLs, which expand into an integrated trans-omics network (**far right**). Machine–learning–based integration is expected to accelerate the construction of accurate, interpretable networks, providing a systems-level representation poised to enhance the interpretation and prediction of bacterial phenotypes. In this image, each color denotes a distinct omics layer: gray represents the genome, blue the transcriptome, orange the proteome, and green the interactome.

Taken together, scalable construction of genetically diverse bacterial libraries coupled with high-throughput phenotyping is beginning to make fine-resolution QTL analysis feasible in bacterial systems. In parallel, high-throughput genome tagging and interaction assays are expanding access to dynamic, in vivo protein–protein interaction maps under native regulation. The protein interactome is one of the omics layers most proximal to cellular function. It is influenced by regulation across the genome, transcriptome, and proteome and includes intrinsic feedback loops [111,145]. Consequently, comprehensive characterization of the interactome requires multi-layer measurements and integrative analytical frameworks [146,165,166]. In eukaryotes, machine-learning approaches have captured cross-omic relationships, revealed new gene functions and regulatory mechanisms [166,167]. As experimental and analytical methods tailored for bacteria mature and datasets grow, these approaches should become more broadly applicable and interpretable, strengthening links between genotype, molecular states, and phenotypes in bacteria (Figure 2).

## 4. Conclusions

Bacterial genetics has evolved from static catalogs of individual molecular characteristics, such as genes, transcripts, and proteins, to a dynamic, system-level analysis of molecular networks. This transformation has been driven by technological and analytical advances across multiple omics fields. Genome-wide association studies (GWAS) and machine learning first enabled the prediction of genotype–phenotype relationships. Transcriptomics and proteomics, particularly at single-cell and spatial resolutions, subsequently revealed dynamic functional states, cellular heterogeneity, and multiple layers of regulatory control. Interactome mapping leverages both experimental approaches, including mass spectrometry-based analyses (AP-MS, PL, and XL-MS) and binary protein–protein interaction detection methods (Y2H, B2H, and PCA), as well as AI-based prediction. These approaches are now uncovering system-level cell mechanisms that enable bacteria to respond to environmental changes, such as host infection or antibiotic exposure.

The ultimate goal of bacterial systems biology is integration. Bacterial multi-omics and quantitative trait locus (QTL) analyses are building a framework that connects these layers, establishing causal links from genetic variants to RNA expression (eQTLs), protein abundance (pQTLs), and ultimately complex phenotypes. This integrative approach enables researchers to trace the full trajectory from subtle genetic variations, such as point mutations, small indels, and chromosomal rearrangements, to system-wide network perturbations. It further links these perturbations to observable traits, including antibiotic resistance, virulence, and host specificity.

Although challenges remain, the field stands at an inflection point. Emerging technologies such as artificial recombination, high-throughput phenotyping, deep mutational scanning, microfluidics, and multiplexed genome editing are converging to enable predictive and dynamic modeling of bacterial life. Protein-fragment complementation assays will facilitate analyzing dynamic interactome regulation under native states, while high-throughput QTL platforms will help elucidate the genetic basis of complex phenotypes.

Together, these synergistic innovations will generate integrated multi-omics datasets that connect genotype to phenotype through intermediate molecular layers. To extend these approaches across diverse bacterial species, species-specific optimization will be required, best supported by international collaboration through shared public databases. As these resources expand and standardize, machine-learning methods can fuse multimodal evidence into interpretable, predictive models of bacterial systems. This system-level perspective will accelerate the development of microbiology by uncovering uncharacterized genomic features and enabling transformative applications, from designing antimicrobials that target network vulnerabilities to engineering microbial cell factories and deciphering ecosystems that shape health and the environment. The long-envisioned transition from SNPs to networks is now well underway, bringing a predictive and systems-level understanding of bacterial life within reach.

## Figures and Tables

**Table 1:** Advances in Bacterial Transcriptomics and Proteomics.

Technology	Layer	Resolution	Key Features	Advantages	Limitations	Ref.
Bulk RNA-seq	Transcriptome	Population	Short-read sequencing of pooled transcripts	High throughput, broad dynamic range	Averages out cell heterogeneity	[69]
Dual RNA-seq	Transcriptome	Population (host + microbe)	Simultaneous sequencing of host and bacterial transcripts	Captures infection dialogue	Complex analysis; host RNA often dominates	[20]
Oxford Nanopre Technology (ONT) Long-Read Sequencing	Transcriptome	Population	Direct sequencing of cDNA or native RNA; maps operons & detects RNA modifications	Preserves modifications; resolves full-length transcripts	Lower throughput; base-calling errors	[72]
Single-cell RNA-seq	Transcriptome	Single-cell	FACS + random-hexamer priming (MATQ-seq); split-pool barcoding (PETRI-seq, MicroSPLiT); droplet-based platforms (M3-seq, BacDrop, smRandom-seq); droplet + probe (ProBac-seq)	Detects extremely rare subpopulations (<0.1%); reveals heterogeneity within clonal populations	Lower throughput; complex workflows; higher cost	[21,22,23,24,70,73,85,88,89]
Spatial transcriptomics (e.g., par-seqFISH)	Transcriptome	Spatial	Sequential hybridization and imaging of marker genes in fixed biofilm	Spatial mapping of expression at micron scale	Limited number of target genes; requires fixed samples	[25]
DIA-MS	Proteome	Population	Systematic fragmentation of all detectable precursor ions	High reproducibility; fewer missing values; quantitative	Requires optimized spectral libraries	[96]
Top-Down Proteomics (TDP)	Proteome	Proteoform	Intact protein analysis to capture sequence variants and PTMs	Direct identification of proteoforms; PTM mapping	Low throughput; specialized equipment	[101,102]
Single-Bacterium Proteomics (SBP)	Proteome	Single-cell	SCOPE-MS with carrier proteome	Detects proteins in individual bacterial cells	Very low protein amounts; method still developing	[104]
EXCRETE Workflow	Proteome	Secretome	Bead-based aggregation & digestion	High-yield, high-throughput secretome profiling	Limited to extracellular proteins; may miss low-abundance targets	[108]

**Table 2:** Methods for Bacterial Interactome Mapping.

Method	Interaction Type	In Vivo/ In Vitro	Resolution	Strengths	Limitations	Ref.
Yeast Two-Hybrid (Y2H)	Binary PPIs	In Vivo (yeast)	Protein–protein	High throughput; well-established; cost-effective	Non-native environment for bacterial proteins; may produce false positives/negatives	[120]
Bacterial Two-Hybrid (B2H)	Binary PPIs	In Vivo	Protein–protein	Native bacterial environment; effective for membrane proteins; high throughput	May miss transient interactions; exogenous system may alter relative abundance of hybrid proteins	[121]
Protein Fragment Complementation Assay (PCA)	Binary PPIs	In Vivo	Protein–protein	Detects interactions under native regulatory control	Requires genome tagging of all target genes; potential labeling bias	[144]
Affinity Purification–MS (AP-MS)	Stable complexes	In Vitro	Complex composition	Quantitative (q-AP-MS); adaptable to many proteins	Requires tagged bait; may disrupt physiological interactions; may miss weak/transient interactions	[126]
Proximity Labeling (PL)	Stable + transient	In Vivo	Spatial proximity (~10–20 nm)	Captures weak/transient interactions; preserves native state	Labeling bias; requires fusion construct; difficult to distinguish between direct/indirect associations	[131]
Cross-Linking MS (XL-MS)	Stable + transient	In Vivo/In Vitro	Residue-level	Provides structural constraints; models large complexes	May miss weak/transient interactions due to cross-linker accessibility; complex workflow	[134,135]
AlphaFold-Multimer	Predicted PPIs	In Silico	Structural model	Proteome-scale predictions; structural insight	Requires experimental validation	[138]

## Data Availability

Not applicable.

## Abbreviations

The following abbreviations are used in this manuscript:

AMR	Antimicrobial resistance
AP-MS	Affinity purification–mass spectrometry
B2H	Bacterial two-hybrid
DBG	de Bruijn graph
DDA	Data-dependent acquisition
DIA	Data-independent acquisition
DRS	Direct RNA sequencing
FACS	Fluorescence-activated cell sorting
GWAS	Genome-wide association studies
LD	Linkage disequilibrium
LMM	Linear mixed models
MDS	Multidimensional scaling
ONT	Oxford nanopore technology
PCA	Principal component analysis
PL	Proximity labeling
PPI	Protein–protein interaction
PTM	Post-translational modification
QTL	Quantitative trait locus
SBP	Single-bacterium proteomics
SNP	Single-nucleotide polymorphism
SVM	Support vector machine
TDP	Top-down proteomics
TRIPs	Transcription–replication interaction profiles
XL-MS	Cross-linking mass spectrometry
Y2H	Yeast two-hybrid
